# A cell-based screening system for anti-influenza A virus agents

**DOI:** 10.1038/srep08672

**Published:** 2015-03-02

**Authors:** Wan Ying Wong, Sheng Wei Loh, Wei Lun Ng, Ming Cheang Tan, Kok Siong Yeo, Chung Yeng Looi, Mohd Jamil Maah, Chee-Kwee Ea

**Affiliations:** 1 Institute of Biological Sciences, Faculty of Science, University of Malaya, 50603 Kuala Lumpur, Malaysia; 2 Department of Pharmacology, Faculty of Medicine, University of Malaya, 50603 Kuala Lumpur, Malaysia; 3 Department of Chemistry, Faculty of Science, University of Malaya, 50603 Kuala Lumpur, Malaysia

## Abstract

Emerging of drug resistant influenza A virus (IAV) has been a big challenge for anti-IAV therapy. In this study, we describe a relatively easy and safe cell-based screening system for anti-IAV replication inhibitors using a non-replicative strain of IAV. A nickel (II) complex of polyhydroxybenzaldehyde N4-thiosemicarbazone (NiPT5) was recently found to exhibit anti-inflammatory activity *in vivo* and *in vitro*. NiPT5 impedes the signaling cascades that lead to the activation of NF-κB in response to different stimuli, such as LPS and TNFα. Using our cell-based screening system, we report that pretreating cells with NiPT5 protects cells from influenza A virus (IAV) and vesicular stomatitis virus (VSV) infection. Furthermore, NiPT5 inhibits replication of IAV by inhibiting transcription and translation of vRNAs of IAV. Additionally, NiPT5 reduces IAV-induced type I interferon response and cytokines production. Moreover, NiPT5 prevents activation of NF-κB, and IRF3 in response to IAV infection. These results demonstrate that NiPT5 is a potent antiviral agent that inhibits the early phase of IAV replication.

Influenza A virus (IAV) belongs to the *Orthomyxoviridae* family. It is an enveloped virus with negative sense RNA segmented genome that encodes for at least 11 viral proteins, namely, hemagglutinin (HA), neuraminidase (NA), matrix 1 (M1), matrix 2 (M2), nucleoprotein (NP), non-structural protein 1 (NSP1), non-structural protein 2 (NS2; also known as nuclear export protein, NEP), polymerase acidic protein (PA), polymerase basic protein 1 (PB1), polymerase basic protein 2 (PB2) and polymerase basic protein 1-F2 (PB1-F2)^1^. IAV has caused pandemics around the world since a century ago, for example, the Spanish flu pandemic in 1918 caused by the IAV subtype H1N1, the Asian flu pandemic in 1957 caused by influenza H2N2, the Hong Kong H3N2 Flu pandemic, the most recent H1N1 outbreak in 2009. The flu pandemics not only lead to economic losses but also become an increasing burden to animal and human health worldwide. Commercially available anti-influenza drugs mainly fall into two major classes: M2 protein inhibitors (amantadine) and neuraminidase inhibitors (oseltamivir)^2^. However, emerging resistance to these inhibitors among the circular seasonal influenza virus highlights the needs to develop new classes of inhibitors^3^.

In the early of 20^th^ century, thiosemicarbazone (TSC) complex was found to have some important biological properties. In the 1950s, TSC was shown to be anti-tuberculosis and anti-leprosy^4^. It was demonstrated as an antiviral agent against vaccinia virus (VV)^5^. A thiosemicarbazone compound, 1-methylisatin 3-thiosemicarbazone or better known as methisazone (brand name: Marboran), was commercialized as an antiviral drug against smallpox infection^6^. TSC exhibits other important biological properties, including antitumor, antiprotozoal, and antibacterial effects^7^. The broad range of thiosemicarbazone bioactivity is closely related with its interaction with different metal ions; the presence of the metal ion complexes on TSC increases the biological effects and mitigates the side effects of the organic parent compound^8^. We previously showed that a Nickel (II) complex of polyhydroxybenzaldehyde N4-thiosemicarbazone (NiPT5) is an anti-inflammation agent by blocking the TNFα- and LPS-induced activation of NF-κB^9^. In addition, NiPT5 suppresses carrageenan-induced paw edema formation in mice.

Here we report a simple and safe cell-based screening system for IAV replication inhibitors and demonstrate the potent antiviral effect of NiPT5 against IAV and vesicular stomatitis virus (VSV).

## Results

### A cell-based screening system for IAV replication inhibitors

**Figure 1:**
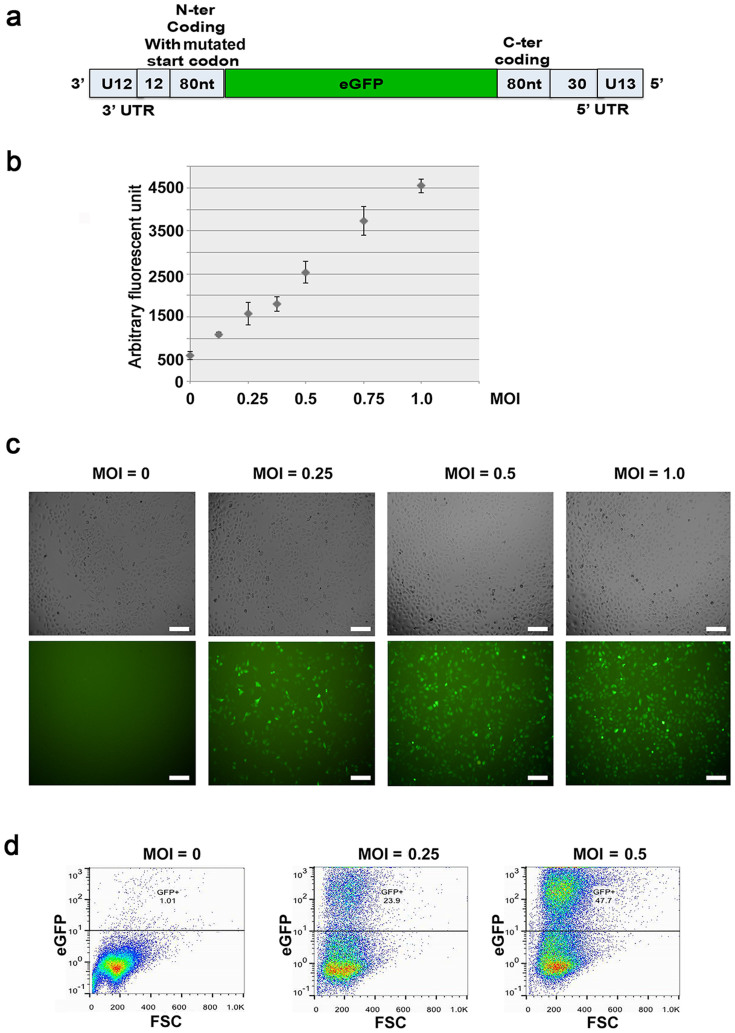
A cell-based screening system for IAV replication inhibitors. (a) PB1Flank-eGFP viral RNA contains the 80 terminal coding nucleotides with mutated start codon flanking by untranslated regions from the PB1 segment. (b) A549-PB1 cells were seeded in a 96 wells plate and infected with different MOI of PR8-PB1flank-eGFP virus (IAV). After 24 hours, cells were stained with ER tracker as an internal control to normalize the eGFP fluorescent signal. The total fluorescent signals were measured with a Tecan Infinite F200 Pro fluorometer (b). Results represent the mean ± SD in quadruplicate experiments. Cells were visually examined on an Olympus IX73 inverted microscope at 200× final magnification and photographed using an Olympus DP73 digital camera and Cellsens standard software (c); or analyzed with FACS (d). Scale: 20 μm.

We adopted a cell-based assay that was originally designed to study the evolution of IAV oseltamivir resistance^10^ as a novel cell-based screening system for IAV replication inhibitors. A non-replicative PR8 strain IAV carrying eGFP in the place of the PB1 gene (PR8-PB1flank-eGFP) (Fig. 1a) was used to infect A549 cells that stably expressing PB1 protein (A549-PB1). The eGFP reporter gene was flanked by minimum sequences required for efficient and stable packaging into virions^10^. Upon PR8-PB1flank-eGFP viral infection and replication, infected A549-PB1 cells will express the eGFP reporter. Thus the IAV infection can be easily monitored and measured by fluorometry (Fig. 1b), fluorescent microscopy (Fig. 1c), and fluorescent-activated cell sorting (FACS) (Fig. 1d). A549-PB1 cells were stained with ER-tracker as an internal control for fluorometry measurement (Fig. 1b). In all cases, the fluorescent signal correlates very well with the amount of PR8-PB1flank-eGFP virus we used for infection.

### Identification of NiPT5 as an antiviral agent

**Figure 2:**
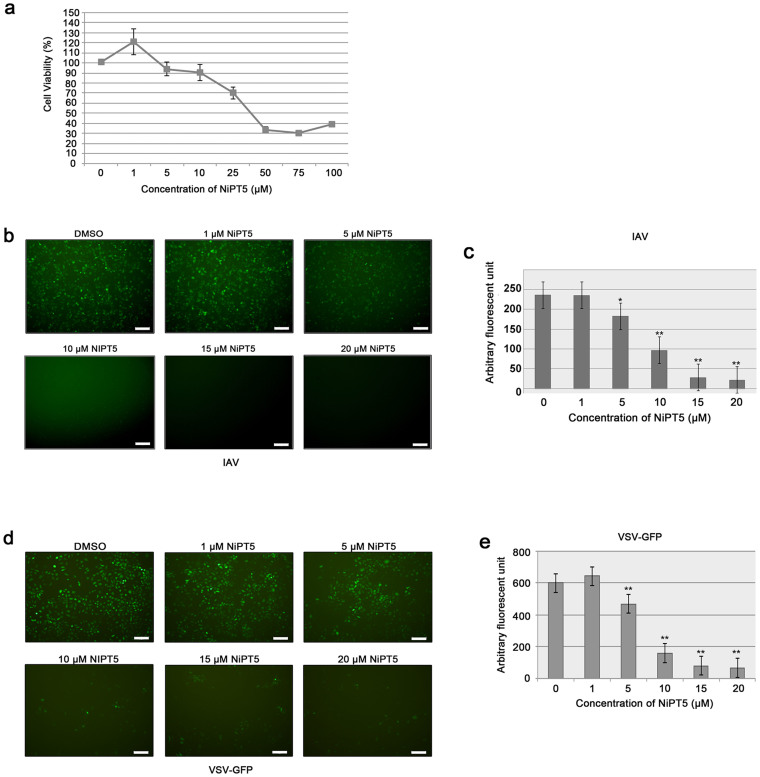
NiPT5 inhibits IAV infection. (a) A549-PB1 cells were cultured with various concentrations of NiTP5 as indicated. After two days, cell proliferation was measured with a MTT assay. Results represent the mean ± SD in quadruplicate experiments. (b) A549-PB1 cells were seeded in a 96 wells plate. A549-PB1 cells were pre-treated with NiPT5 at various concentrations for 4 hours prior infecting the cells with PR8-PB1flank-eGFP virus (IAV) at a MOI 1.0. After 24 hours, cells were stained with ER tracker and analyzed as in Fig. 1, microscopy (b); fluorometry (c). (d–e) A549-PB1 cells were treated as in (b–c), except that VSV-GFP virus was used for infection. Results represent the mean ± SD in quadruplicate experiments. Student's t-test: *, p < 0.05; **, p < 0.01. Scale: 20 μm.

We previously showed that NiPT5 inhibits TNF- and LPS-induced activation of NF-κB^9^. Induction of IFNβ upon virus infection requires the activation of NF-κB^11^. Thus, NiTP5 may render cells sensitive to virus infection and is an ideal compound to test our cell-based screening system. Prior to understand the effect of NiPT5 on IAV infection, we determined the cytotoxic effect of NiPT5 in A549-PB1 cells with a standard MTT assay. After two days incubation, the IC_50_ of NiPT5 was 45.3 μM in A549-PB1 cells (Fig. 2a).

To test if NiPT5 renders cells sensitive to virus infection, we pretreated A549-PB1 cells with different concentrations of NiPT5 for four hours and infected the cells with PR8-PB1flank-eGFP virus at a multiplicity of infection (MOI) of 1.0. Pre-treating the A549-PB1 cells with NiPT5 did not render the cells more sensitive to IAV infection (Fig. 2b and 2c). In contrary, NiPT5 protected the cells from IAV infection in a dose dependent manner. Pretreating A549-PB1 cells with 5 μM of NiPT5 reduced GFP positive cells (Fig. 2b) and significantly decreased the total fluorescent signal by 23% (Fig. 2c p = 0.036). IAV infection was completely abolished at 15 μM or higher concentration of NiPT5. The antiviral activity of NiPT5 was not due to the cytotoxicity of NiPT5, since cell proliferation was unaffected at 5 μM and only slightly decreased at 10 μM (Fig. 2a and S1a). Furthermore, we showed that NiPT5 was working in A549-PB1 cells since it inhibited TNF-induced activation of NF-κB in A549-PB1 cells (Fig. S1b). Pretreating A549-PB1 cells with NiPT5 dramatically inhibited the induction of several NF-κB target genes, including *IP10*, *CCL5* and *IL1β* in response to TNFα stimulation. These results suggest that NiPT5 shows antiviral activity against IAV and our cell-based provides a quick and easy way to screen for anti-IAV agents.

To test if NiPT5 exerts antiviral activity against other virus, we pretreated A549-PB1 cells with NiPT5 for four hours and infected A549-PB1 cells with a vesicular stomatitis virus carrying a GFP (VSV-GFP) at a MOI of 1.0. In the DMSO treated control A549-PB1 cells, VSV-GFP infection resulted in greater than 90% of GFP positive cells (Fig. 2d). NiPT5 reduced GFP positive cells (Fig. 2d and S1c) and the total fluorescent signal (Fig. 2e) in a dose-dependent manner. This finding suggests that NiPT5 protects cells from VSV-GFP infection.

### NiPT5 reduces IAV replication

**Figure 3:**
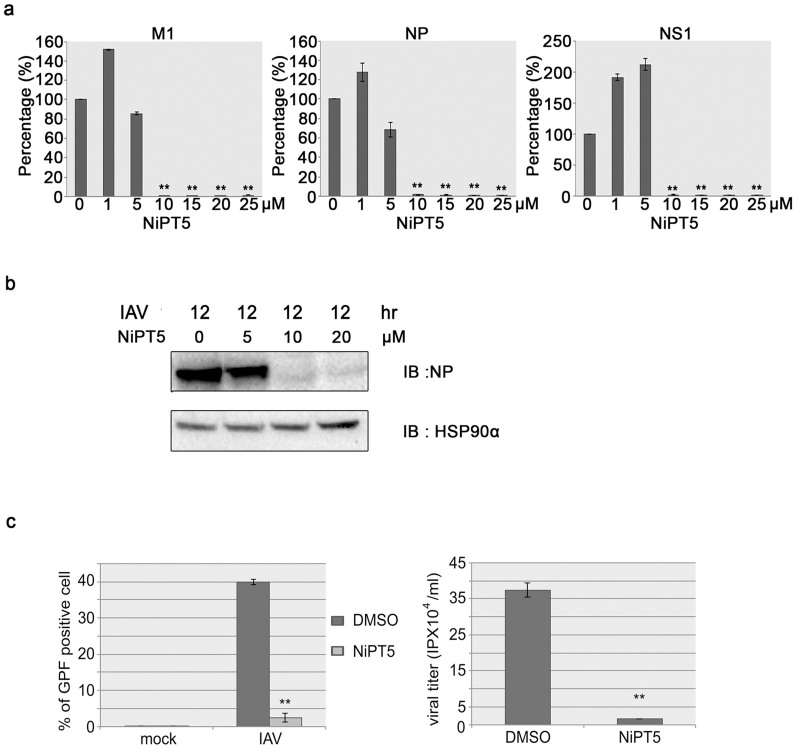
NiPT5 prevents the replication of influenza virus. (a) A549-PB1 cells were treated with various concentrations of NiPT5 as indicated four hours prior to infecting the cells with PR8-PB1flank-eGFP virus (IAV) at a MOI 1.0. After twelve hours, the expressions of virus NP, M1 and NS1 were measured with RT-qPCR. Error bars represent the variation range of duplicate experiments. (b) The A549-PB1 cells were treated as in (a), whole cell extracts were prepared and were subjected to immunoblotting with antibodies against NP and HSP90. Full-length blot is presented in Supplementary Figure S4a. (c) A549-PB1 cells were pre-treated with 10 μM NiPT5 for four hours prior to infecting the cells with IAV virus at a MOI 0.5. After twenty hours, infected cells were analyzed with FACS (left panel) while the supernatant was harvested and subjected to viral titering assay using FACS (right panel). IP: infectious viral particle. Student's t-test: **, p < 0.01.

To test whether NiPT5 inhibits viral replication, we investigated IAV polymerase activity by measuring the expression of viral RNAs (vRNAs) with RT-qPCR. A549-PB1 cells were pretreated with NiPT5 at various concentrations for four hours and were then infected with PR8-PB1flank-eGFP virus at a MOI of 1.0. The expression of influenza vRNAs, NP, NS and M1, were measured with RT-qPCR twelve hours post-infection. The expression of NP, NS and M1 was readily detected in PR8-PB1flank-eGFP virus infected DMSO-treated control A549-PB1 cells (Fig. 3a). On the other hand, NiPT5 inhibited the transcription of NP, NS and M1 in a dose-dependent manner. For example, at the concentration of 10 μM of NiPT5, the expression of NP, NS and M1 was reduced by 98%. To determine whether NiPT5 affects the influenza virus protein level, whole cell extracts were prepared twelve hours post-infection and the NP protein level was measured by immunoblotting with antibody against NP protein. NP protein was detected in PR8-PB1flank-eGFP virus infected A549-PB1 cells but not detected in the presence of 10 μM of NiPT5 (Fig. 3b). This result indicates that NiPT5 blocks viral protein synthesis.

To explore whether NiPT5 influences the replication of virus, we measured viral titer in the culture media collected from PR8-PB1flank-eGFP virus infected A549-PB1 cells in the presence or absence of NiPT5. We quantified infected cells by sorting the virus-infected, eGFP-positive cells using FACS. Consistent with fluorescent microscopy analysis (Fig. 2b), NiPT5 considerably inhibited IAV infection (Fig. 3c, left panel). Next, supernatants from infected A549-PB1 cells were collected and subjected to virus titering assay using FACS. We found that there was about 20-fold reduction of new virus generated in the presence of NiPT5 (Fig. 3c, right panel). Taken together these results suggested that NiPT5 inhibits IAV replication.

### NiPT5 inhibits IAV-induced interferon and cytokine expression

**Figure 4:**
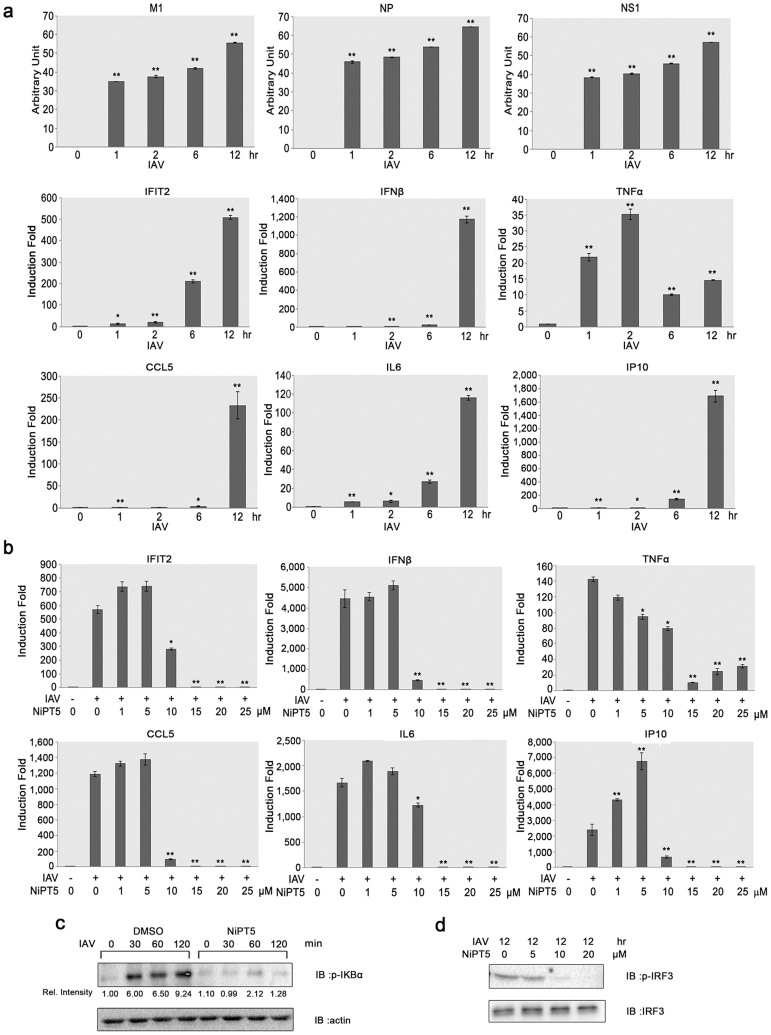
NiPT5 inhibits IAV induced ISGs and cytokines expression. (a) A549-PB1 cells were infected with PR8-PB1flank-eGFP virus (IAV) at a MOI 1.0. The expression of vRNAs, ISGs and cytokines was measured with RT-qPCR at the indicated time periods. (b) A549-PB1 cells were treated with various concentrations of NiPT5 as indicated four hours prior to infecting the cells with IAV at a MOI 1.0. After twelve hours, the expression of various ISGs and cytokines was measured with RT-qPCR. Error bars represent the variation range of duplicate experiments. *: p < 0.05; **: p < 0.01. (c–d) Whole cell extracts were prepared from A549-PB1 cells as in (b), and examined by immunoblotting using antibodies against p-IκBα, Rel. Intensity: intensity of bands was quantified using the Image Lab (BioRad) software and was normalized to actin (c), p-IRF3 (d). Full-length blots are presented in Supplementary Figure S4.

Upon viral infection, host cells will mount the type I interferon response and produce cytokines to induce innate immunity against virus. To test if NiPT5 inhibits virus infection through enhancement of antiviral responses in host cells, we measured the expression of type I interferon response genes (ISGs) and cytokines upon IAV infection with or without pretreating A549-PB1 cells with NiPT5. First we checked the gene expression kinetics of several vRNAs, ISGs and cytokines under our experiment setting. We infected A549-PB1 cells with PR8-PB1flank-eGFP virus at a MOI 1.0 and measured gene expression with RT-qPCR at the indicated time points. The expression of vRNAs was detected as early as 1 hour post-infection (Fig. 4a). Most of the ISGs and cytokines, including *IFNβ*, *IFIT2*, *TNFα*, and *IL6* were detected two hours post-infection. The expression of late genes, *IP10* and *CCL5*, was detected twelve hours post-infection. The expression of ISGs and cytokines at twelve hours post-infection was substantially reduced in the presence of 10 μM of NiPT5 and was abolished in 15 μM of NiPT5 treated cells (Fig. 4b). These results imply that NiPT5 does not protect cells from IAV infection by enhancing antiviral responses in the host cells.

### NiPT5 inhibits IAV induced NF-κB and IRF3 activation

NF-κB and IRF3 pathways mainly regulate the production of virus-induced ISGs and cytokines. To examine if NiPT5 inhibits IAV induced NF-κB and IRF3 activation, we measured the phosphorylation of IκBα (p-IκBα) and the phosphorylation of IRF3 (p-IRF3), a biochemical hallmark of NF-κB and IRF3 activation respectively. The p-IκBα was detected at 30 minutes post-infection (Fig. 4c), while the p-IRF3 was detected twelve hours post-infection (Fig. 4d and S2). Pretreating A549-PB1 cells with NiPT5 greatly reduced IAV-induced phosphorylation of IκBα (Fig. 4c). Furthermore, IAV-induced phosphorylation of IRF3 was inhibited by NiPT5 in a dose-dependent manner (Fig. 4d). These results suggest that NiPT5 inhibits IAV-induced activations of NF-κB and IRF3.

## Discussion

NiPT5 was first characterized as a potent anti-inflammation agent by inhibiting activation of NF-κB induced by TNFα and LPS stimulations^9^. In this study, NiPT5 shows antiviral activity against IAV and VSV (Fig. 2). NiPT5 inhibits the transcription of IAV vRNAs and the expression of IAV NP protein (Fig. 3). Moreover, NiPT5 does not protect the A549-PB1 cells from virus infection by enhancing the intrinsic antiviral response in the host cells. NiPT5 however inhibits virus-induced activation of NF-κB and IRF3 (Fig. 4).

Type I interferon response is the key intrinsic antiviral pathway blocking type I interferon response will render cells susceptible to virus infection^12^. We observed that NiPT5 inhibits type I interferon response but does not sensitize cells to virus infection suggesting that NiPT5 may block viral replication at a step before the detection of virus infection by the host cells. IAV is detected by host cells through TLRs, RIG-I, and NLRP3 pathways^13^. Its genomic ssRNA is detected by TLR7/8 in the lumen of endosome^14^,^15^, and by RIG-I in the cytosol^16^. NLRP3 is activated through influenza M2 mediated disturbances of intracellular ionic concentrations^17^. A549 cells do not express TLR7/8^18^ and NLRP3 does not induce type I interferon response^19^. As a result, detection of influenza virus infection by A549-PB1 cells mainly relies on the RIG-I pathway. The activation of RIG-I requires the release of influenza genomic ssRNA from endosome to cytoplasm. Thus NiPT5 may inhibit IAV replication at or before the release of influenza genomic ssRNA from the endosome, including attachment of virus particle to cell surface receptor or internalization through virus-host cell fusion. However, we cannot rule out the possibility that NiPT5 inhibits both IAV replication as well as IAV-induced IRF3 and NF-κB activation. Further experiments are required to understand the molecular mechanism of NiPT5-mediated antiviral infection.

In conclusion, we succeeded in using a nonreplicative PR8 strain of influenza virus carrying eGFP in the place of the PB1 gene (PR8-PB1flank-eGFP) and A549-PB1 cells to demonstrate the antiviral activity of NiPT5. The PR8-PB1flank-eGFP virus is lacking the PB1 gene and thus will not replicate in normal cells. With the incorporation of an eGFP reporter, the infected cells can be quantified either using FACS or a fluorometer. This system is suitable for screening inhibitors that block IAV replication from any step of the viral life cycle. Moreover, this system can be adopted for high throughput screening for anti-influenza virus agents with the incorporation of an automated liquid handling system together with a fluorometer or an automated fluorescent microscope or a FACS.

## Methods

### Cell culture

A549-PB1 cells and 293T-PB1 cells were cultured in DMEM supplemented with 10% fetal bovine serum (FBS), penicillin G (100 U/ml), and streptomycin (100 μg/ml).

### Antibodies and compounds

Antibodies against actin (Santa Cruz Biotech), Hsp90α (Santa Cruz Biotech), p-IκBα (Cell Signaling), p-IRF3 (Cell Signaling) and NP (H16-L10-4R5, ATCC) were purchased from the respective commercial sources. NiPT5 was synthesized as described previously^9^. NiPT5 was dissolved in DMSO and DMSO was used as a mock treatment.

### Cell proliferation assay

A549-PB1 cells were treated with or without various concentrations of NiPT5 in a 96 wells format. After incubation for two days, 10 μl of 2 mg/ml 3-(4,5-dimethylthiazol-2-yl)-2,5-diphenyltetrazolium bromide (MTT) in DMEM medium was added and cells were further incubated for three hours at 37°C in a CO_2_ incubator. Cells were spun down at 2500 rpm for 5 minutes and the medium was carefully removed. One hundred and fifty microliters of DMSO was added to each well. After pipetting up and down several times, the absorbance was measured with a M200 PRO microplate reader (Tecan) at the wavelength of 540 nm. IC_50_ was calculated with GraphPad Prism 6.

### RT-PCR

A549-PB1 cells pretreated with or without NiPT5 for 4 hours were infected with PR8-PB1flank-eGFP virus at a multiplicity of infection (MOI) of 1.0 for 12 hours. Total RNAs were isolated with the Thermo Scientific GeneJET RNA Purification Kit. Complementary DNAs were synthesized and Quantitative PCR was performed with 2X SYBR Green PCR Master mix (Thermo Scientific) and run on a Bio-Rad Connect Real-Time PCR System. All data were then normalized to L32. The sequences of the primers are listed in table S1.

### Virus amplification

PR8-PB1flank-eGFP virus was amplified by infecting 239T-PB1 cells. Prior to virus infection, 293T-PB1 cells were washed once with PBS and then changed into influenza growth media (RPMI supplemented with 0.2% bovine serum albumin, 0.01% heat-inactivated fetal bovine serum, 100 U/ml penicillin, 100 μg/ml streptomycin and 1 mM calcium chloride) containing 2 μg/ml TPCK-treated trypsin. After 48 hours the supernatant was harvested through a 0.45 μm filter. The virus titer was determined by FACS (see below).

### Virus infection

Ten thousand A549-PB1 cells per well were seeded in a 96-well flat plate. After overnight incubation, cells were washed once with PBS and then changed into influenza growth media containing 0.2 μg/ml TPCK-treated trypsin. PR8-PB1flank-eGFP virus was added at a MOI of 1.0. After 24 hours, cells were analyzed with an Olympus IX73 inverted microscope at 200× final magnification and photographed using the Olympus DP73 digital camera and Cellsens standard software. The total fluorescent signal was measured with a Tecan Infinite F200 Pro fluorometer.

Ten thousand A549-PB1 cells per well were seeded in a 96-well flat plate. After overnight incubation, the cells were pre-treated with different concentrations of NiPT5 as indicated for 4 hours and then infected with vesicular stomatitis virus (VSV) carrying a GFP (VSV-GFP) at a MOI of 1.0 for 14 hours.

Cells were also stained with 5 μM ER-tracker Red (Life technology) according to manufacturer's protocol. The total fluorescent signal was measured with a Tecan Infinite F200 Pro fluorometer, and used as an internal control to normalize eGFP or GFP fluorescent readings.

### Virus titering

Virus titer was determined as previous described with slight modification^10^. Briefly, A549-PB1 cells were pretreated with or without 10 μM NiPT5 for 4 hours and then infected with PR8-PB1flank-eGFP virus at a MOI of 0.5 in influenza growth media plus 2 μg/ml TPCK-treated trypsin. Twenty-four hours later the supernatants were collected and filtered with 0.45 μm filters. The viruses in the collected supernatants were tittered by infecting fresh A549-PB1 cells in influenza growth media plus 0.2 μg/ml TPCK-treated trypsin. Low concentration of TPCK-treated trypsin was used to ensure no infectious IAV was produced to avoid any secondary infection (Figure S3). After 24 hours, cells were washed with PBS and fixed with 0.1% formaldehyde to inactivate virus. Fixed cells were analyzed using a MACSQuant Analyzer (Miltenyi Biotec) to quantified eGFP positive cells. Data were further analyzed with FlowJo software. Total infectious virus particle (IP) was calculated based on the following formula: IP/ml = (% of eGFP positive cell) X (total cell number)/(total volume of supernatant used to infect the cells, in ml). One IP per cell was used when infecting cells at MOI of 1.0.

### Immunoblotting

Two hundred thousand A549-PB1 cells were seeded. After overnight incubation, culture media were removed and changed into IAV infection media. The cells were pre-treated with different concentration of NiPT5 for 4 hours followed by PR8-PB1flank-eGFP virus infection at a MOI of 1.0 for 12 hours. Whole cell lysate extracts were prepared from treated cells using a kinase lysis buffer (20 mM Tris pH7.5, 120 mM NaCl, 10% glycerol, 1% Triton X-100, 25 mM β-glycerophosphate, 1 mM sodium orthovanadate, 1 mM DTT, and 1 mM PMSF). After 30 minutes incubation on ice, lysates were collected by centrifugation at 15 k rpm 4°C for 10 minutes. Immunoblotting was performed using anti-p-p38, anti-NP, anti-p-IRF3, and anti-HSP90α antibodies. Western blot images were captured using ChemiDoc™ MP System (Bio-Rad). Images were processed using BioRad Image Lab version 5.0 and Adobe Photoshop CS4.

### Statistical analysis

Data were analyzed by Microsoft Excel and presented as the mean ± SD. Data are representative of three or more independent experiments. Statistical significance was assessed by use of the Student's t-test.

## Author Contributions

W.Y.W., S.W.L., W.L.N., M.C.T., K.S.Y. and C.-K.E. performed the experiments. C.Y.L. and M.J.M. synthesized NiPT5. C.-K.E. designed experiments, and analyzed the data. C.-K.E. and W.Y.W. wrote the manuscript.

## Supplementary Material

Supplementary Information supplementary info
