# Supplementary material for: A cell-based screening system for anti-influenza A virus agents
Source: Sci Rep. 2015 Mar 2;5:8672. doi: 10.1038/srep08672 (PMC4345322; doi:10.1038/srep08672)
Supplement: Supplementary Information — supplementary info [file srep08672-s1.doc]

A cell-based screening system for anti-influenza A virus agents

Wan Ying Wong, Sheng Wei Loh, Wei Lun Ng, Ming Cheang Tan, Kok Siong Yeo Chung Yeng Looi, Mohd Jamil Maah and Chee-Kwee Ea*

*To whom correspondence should be addressed:

eacheekwee@um.edu.my

Phone: 603-79677022ext2587, Fax: 603-79557727

**Table S1: Primer list**

| hL32/5’ | AGCTCCCAAAAATAGACGCAC |
| --- | --- |
| hL32/3' | TTCATAGCAGTAGGCACAAAGG |
| hIFIT2/5' | GCGTGAAGAAGGTGAAGAGG |
| hIFIT2/3' | GCAGGTAGGCATTGTTTGGT |
| hIFNβ/5’ | ACTGCCTCAAGGACAGGATG |
| hIFNβ/3' | AGCCAGGAGGTTCTCAACAA |
| hIL1β/5' | ACAGATGAAGTGCTCCTTCCA |
| hIL1β/3' | GTCGGAGATTCGTAGCTGGAT |
| hTNFα/5' | GCCCAGGCAGTCAGATCATCT |
| hTNFα/3' | TTGAGGGTTTGCTACAACATGG |
| hCCL5/5' | CCCAGCAGTCGTCTTTGTCA |
| hCCL5/3' | TCCCGAACCCATTTCTTCTCT |
| hIL6/5' | TACCCCCAGGAGAAGATTCC |
| hIL6/3' | TTTTCTGCCAGTGCCTCTTT |
| hIP10/5' | CTGACTCTAAGTGGCATT |
| hIP10/3' | TGATGGCCTTCGATTCTG |
| M1/IAV/5’ | AAGACCAATCCTGTCACCTCTGA |
| M1/IAV/3’ | CAAAGCGTCTACGCTGCAGTCC |
| PR8-NP/IAV/5’ | ACGGCTGGTCTGACTCACAT |
| PR8-NP/IAV/3’ | TCCATTCCGGTGCGAACAAG |
| PR8-NS1/IAV/5’ | AGCAGATAGTGGAGCGGATT |
| PR8-NS1/IAV/3’ | GTACAGAGGCCATGGTCATT |

**Supplementary Figures and legends:**

**
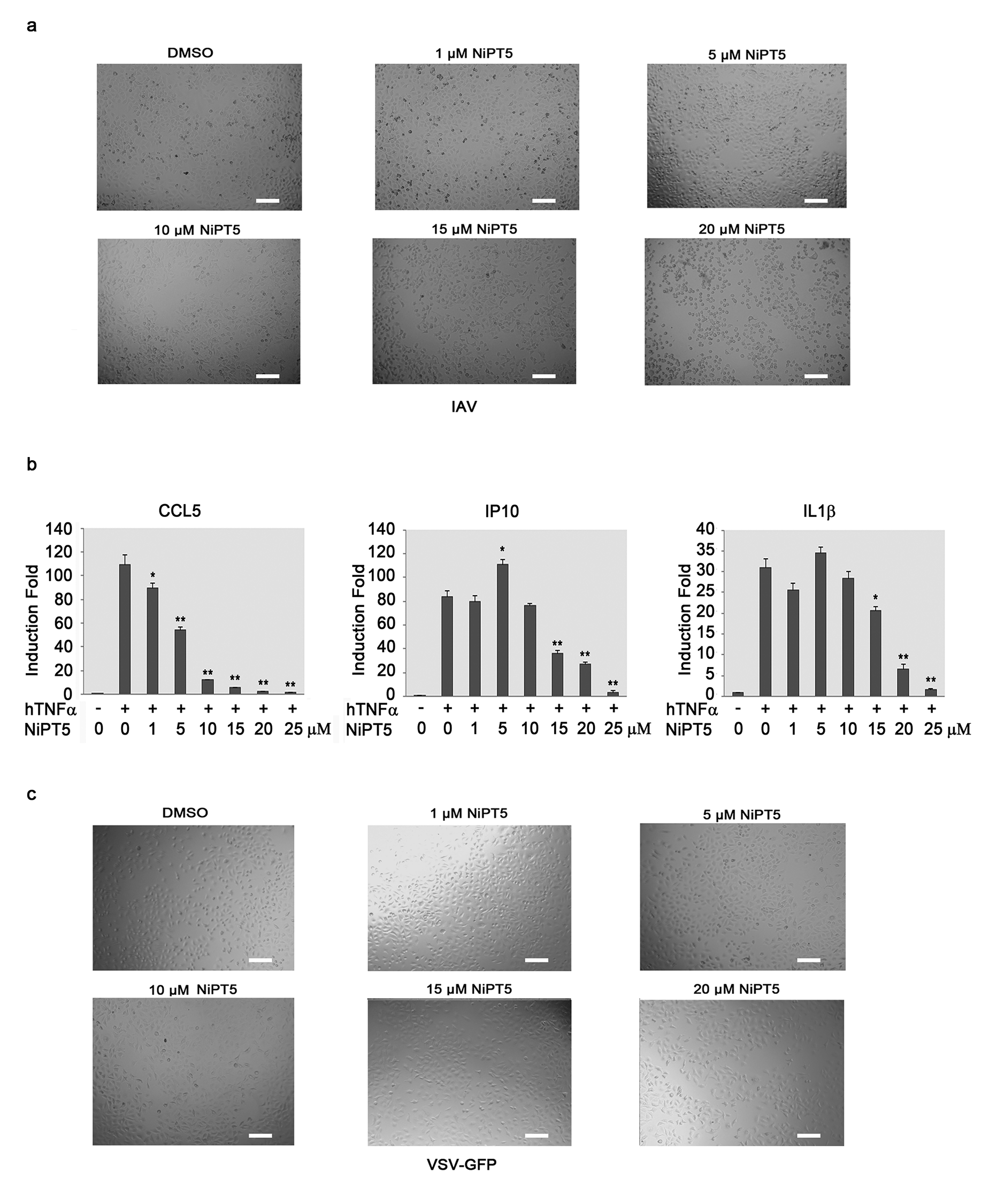
**

**Figure S1. NiPT5 inhibits TNF-induced expression of NF-κB target genes.** (**a**) Bright field images of Figure 2b. Scale: 20 μm. (**b**) A549-PB1 cells were treated with various concentrations of NiPT5 as indicated four hours prior to treating the cells with 10 ng/ml TNFα. After two hours, the expression of *CCL5, IP10* and *IL1β* was measured with RT-qPCR. Error bars represent the variation range of duplicate experiments. *: p < 0.05; **: p < 0.01. (**c**) Bright field images of Figure 2d. Scale: 20 μm.


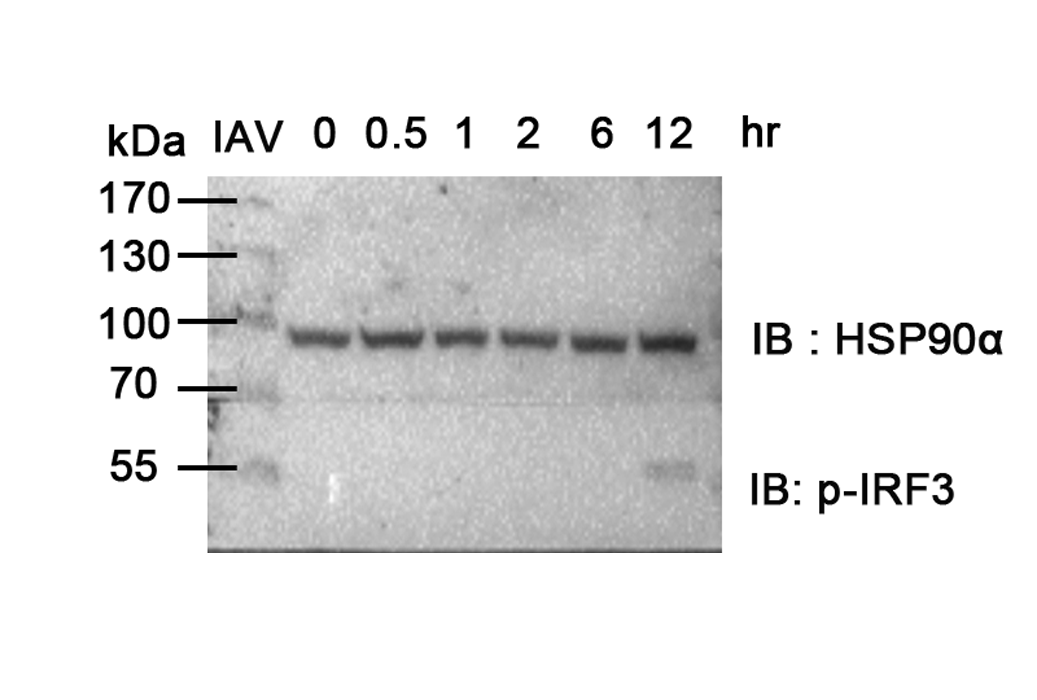


**Figure S2. IAV-induced phosphorylation of IRF3.** A549-PB1 cells were infected with PR8-PB1flank-eGFP virus (IAV) at a MOI 1.0 for the indicated time periods. Whole cell extracts were prepared and examined by immunoblotting using antibodies against p-IRF3 and HSP90α.


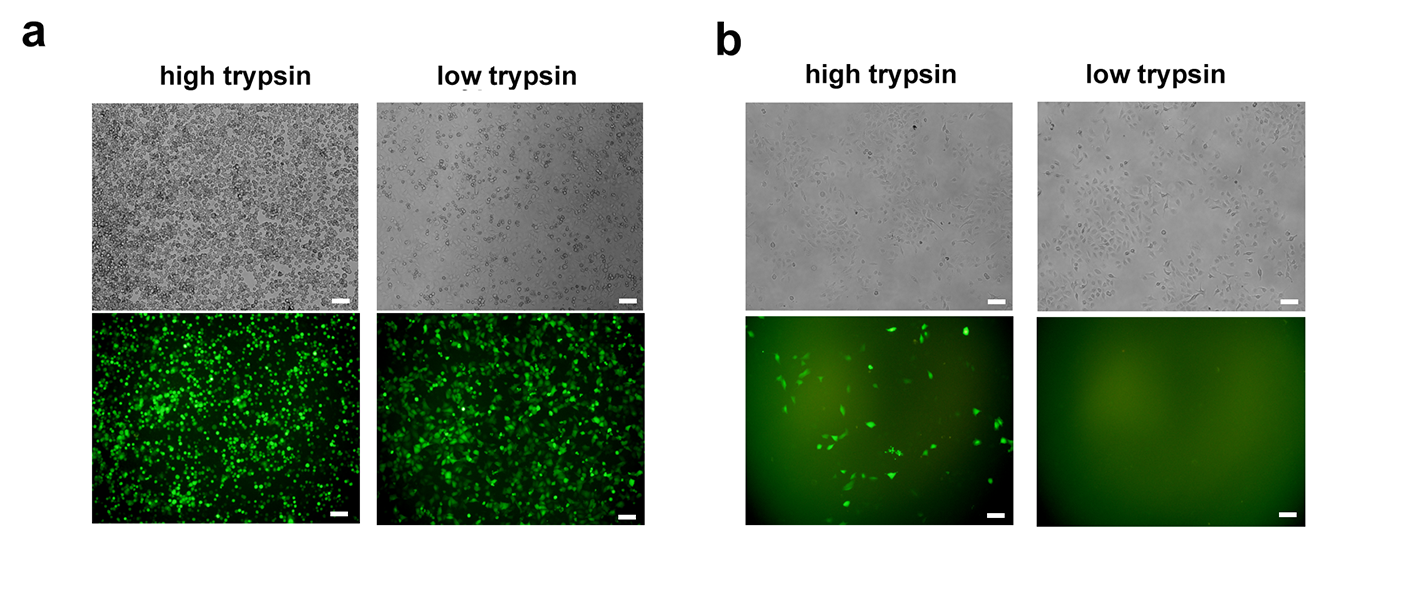


**Figure S3. IAV growth media containing Low TPCK-treated trypsin produced no infectious IAV.** (**a**) Five Hundred thousand A549-PB1 cells were seeded in a 12 well plate and infected with IAV at a MOI of 0.5 in a IAV growth media containing either 2 μg/ml TPCK-treated trypsin (left panel) or 0.2 μg/ml TPCK-treated trypsin (right panel). After 24 hours, cells were visually examined on an Olympus IX73 inverted microscope at 200X final magnification and photographed using an Olympus DP73 digital camera and Cellsens standard software. (**b**) Supernatants from (**a**) were collected and filtered with 0.45 μm filters. Twenty microliter of supernatants were used to infect fresh 100 k A549-PB1 cells seeded in a 12 well plate. After 24 hours, cells were analyzed as in (**a**). Scale: 10 μm.


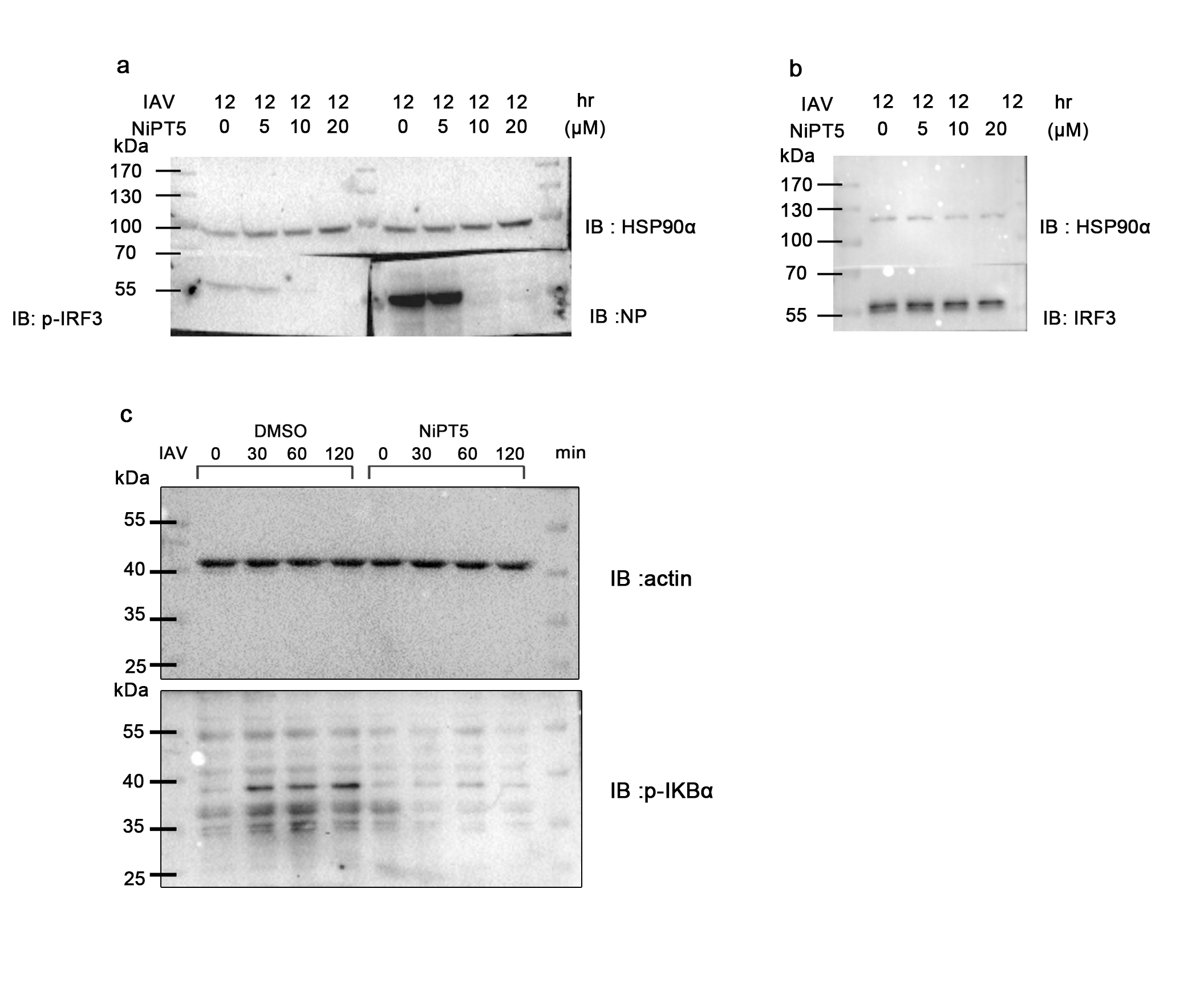


**Figure S4. Full length blots for NiPT5 inhibits IAV replication and infection.** (**a**) Full length blot for Figure 3b (NP) and 4d (p-IRF3) (**b**) Full length blot for Figure 4d (IRF3) (**c**) Full length blot for Figure 4c (p-IκBα). All blots presented are overlay of white light and chemi-luminescent images.
